# A Novel Tetramethylpyrazine Derivative Protects Against Glutamate-Induced Cytotoxicity Through PGC1α/Nrf2 and PI3K/Akt Signaling Pathways

**DOI:** 10.3389/fnins.2018.00567

**Published:** 2018-08-15

**Authors:** Haiyun Chen, Jie Cao, Zeyu Zhu, Gaoxiao Zhang, Luchen Shan, Pei Yu, Yuqiang Wang, Yewei Sun, Zaijun Zhang

**Affiliations:** ^1^Institute of Biomedical and Pharmaceutical Sciences, Guangdong University of Technology, Guangzhou, China; ^2^Institute of New Drug Research and Guangzhou Key Laboratory of Innovative Chemical Drug Research in Cardio-Cerebrovascular Diseases, Jinan University College of Pharmacy, Guangzhou, China

**Keywords:** ischemic stroke, tetramethylpyrazine derivative **22a**, excitotoxicity, neuroprotection, peroxisome proliferator-activated receptor gamma coactivator 1-alpha

## Abstract

Glutamate-induced excitotoxicity is one of the main causes of neuronal cell death in stroke. Compound **22a** has been previously reported as a promising neuroprotective compound derived from tetramethylpyrazine, which is a widely used active ingredient of traditional Chinese medicine Chuanxiong (Ligusticum wallichii Franchat). Compound **22a** can protect neurons from oxidative stress-induced PC12 cell death and alleviates the infarct areas and brain edema in a rat permanent middle cerebral artery occlusion model. In the current work, we further investigated the neuroprotective effects and underlying mechanisms of compound **22a** against glutamate-induced excitotoxicity in primary culture of rat cerebellar granule neurons (CGNs). We found that pretreatment with compound **22a** prevented glutamate-induced neuronal damage by maintaining mitochondrial membrane potential and attenuating cellular apoptosis. Compound **22a** could also enhance peroxisome proliferator-activated receptor gamma coactivator 1-alpha (PGC1α) transcriptional activity and induce nuclear accumulation of Nrf2 in PC12 cells. Accordingly, pretreatment with compound **22a** reversed the glutamate-induced down-regulation of expression of the proteins PGC1α, transcriptional factor NF-E2-related factor 2 (Nrf2), and hemooxygenase 1 (HO-1). In addition, compound **22a** increased the phosphorylation of phosphoinositide 3-kinase (p-PI3K), phosphorylated protein kinase B (p-Akt), and glycogen synthase kinase 3β (p-GSK3β). Meanwhile, the small interfering RNA-mediated silencing of PGC1α expression and selective inhibitors targeting PI3K/Akt (LY294002 and Akt-iv) could significantly attenuate the neuroprotective effect of compound **22a**. Taken together, compound **22a** protected against glutamate-induced CGN injury possibly in part through regulation of PGC1α/Nrf2 and PI3K/Akt pathways.

## Introduction

Ischemic stroke is one of the major causes of human death and disability worldwide (Donnan et al., 2008). Glutamate-induced excitotoxicity has been demonstrated to be involved in neuronal cell death in stroke (Lai et al., 2014). Physiologically, glutamate acts as one of the main excitatory neurotransmitters in the central nervous system (CNS), contributing to normal neural transmission, development, differentiation, and plasticity. Under pathological conditions, however, overproduction of extracellular glutamate leads to uncontrolled, continuous depolarization of neurons in a toxic process called excitotoxicity. Glutamate-induced excitotoxicity is associated with the over-stimulation of glutamate receptors, inducing the impairment of intracellular Ca^2+^ homeostasis and subsequently leading to overproduction of free radicals, overactivation of proteases and kinases, etc. (Wang and Qin, 2010; Lai et al., 2014). Particularly, the overloading intracellular Ca^2+^ and overproduction of free radicals have been shown to induce mitochondrial dysfunction by down-regulating PGC1α, which plays a protective role against neurodegenerative conditions (Sano and Fukuda, 2008; Wareski et al., 2009). Although great progress has been made, the exact mechanism underlying glutamate-induced cytotoxicity is still not clear. Nonetheless, it has been reported that dysregulation of PI3K/Akt and Nrf2 signaling pathways contributes to glutamate-induced excitotoxicity (Jing et al., 2012; Pang et al., 2016). Stimulation of the PI3K/Akt pathway is neuroprotective against hypoxic and excitotoxic neuronal death *in vitro* and ischemic neuronal death *in vivo*, and there is increasingly evidence to indicate cross talk between the Nrf2 and PI3K/Akt pathways in response to glutamate caused cell injury (Jo et al., 2012; Lee et al., 2015).

Since the exact causes of ischemic stroke have yet to be elucidated, currently there are no pharmacological treatments to ameliorate glutamate excitotoxicity and provide neuroprotection for brain ischemic stroke (Lau and Tymianski, 2010). Thrombolysis via the intravenous (i.v.) administration of recombinant tissue plasminogen activator remains the only treatment currently available for acute ischemic stroke. In addition, it is of great concern that clinical trials investigating neuroprotective agents for the development of new stroke therapies have generally been unsuccessful (Grupke et al., 2014). As a result, numerous researchers have been exploring potentially active plant-derived agents, hoping to meet this unmet need and discover a disease-modifying drug (Zhang et al., 2014). Traditional Chinese medicine focuses on the overall regulation of the pathophysiological condition of the entire body, a trait that makes these compounds particularly promising in the treatment of complex diseases (Ghosh et al., 2014). TMP is one of the mainly biologically active constituents derived from the traditional Chinese medicine Chuanxiong (Ligusticum wallichii Franchat) and has been widely used to treat cardio- and cerebro-vascular diseases in clinic (Liu et al., 2003; Xue et al., 2011). In our previous study, a TMP-derived compound **22a** was designed to combine caffeic acid (Touaibia et al., 2011) (another natural compound with versatile pharmacological activities) and a nitrone group (Floyd et al., 2013) (a strong free radical-trapping agent) with TMP. We found that compound **22a** exhibited strong ROS scavenging activity and exerted protective effects in models of ischemic stroke *in vivo* (Chen et al., 2017). In the present study, the neuroprotective effects of compound **22a** against glutamate-induced excitotoxicity on primary culture of rat CGNs, and the underlying mechanisms of action, were further investigated.

## Materials and Methods

### Chemicals and Reagents

All media and supplements used for cell cultures were purchased from Gibco (Carlsbad, CA, United States), unless otherwise noted. ATP assay kit was obtained from Beyotime (Beyotime, China). LY294002 and Akt-iv was obtained from Sigma-Aldrich (St. Louis, MO, United States). PGC1α siRNA, scrambled siRNA and transfection reagent were purchased from Santa Cruz Biotechnology (Santa Cruz, CA, United States). RIPA lysis buffer, phenylmethanesulfonyl fluoride (PMSF), and halt phosphatase inhibitor cocktail were purchased from Pierce Biotechnology (Rockford, IL, United States). Antibodies against phospho-Ser473 Akt, phospho-Ser9 GSK3β, Bcl-2 and Bax were obtained from Cell Signaling Technology (Beverly, MA, United States). Antibodies against β-actin, PGC1α, Nrf2 and HO-1 were obtained from Santa Cruz Biotechnology (Santa Cruz, CA, United States). All other reagents were from Sigma-Aldrich (St. Louis, MO, United States) except where stated otherwise.

### Primary Cell Cultures

Eight-day-old Sprague-Dawley rats were obtained from the Experimental Animal Center of Sun Yat-sen University. The detailed methodology used to separate the CGNs is described in our previous publication (Chen H.Y. et al., 2015). Briefly, neurons were seeded at a density of 1.0–1.5 × 10^5^ cells/well in basal modified Eagle’s medium supplemented with 10% fetal bovine serum, 25 mM KCl, 2 mM glutaMax and penicillin (100 U/mL)/streptomycin (100 μg/mL). The cultures were grown at 37°C for 24 h. The growth of non-neuronal cells was limited by adding cytosine arabinoside (10 μM). Using this protocol, 95–99% of the cultured cells were granule neurons. All experiments were performed in CGNs at 8 days *in vitro* (DIV).

All experiments were conducted in accordance with the guidelines of the Experimental Animal Care and Use Committee of Jinan University. The experimental protocols were approved by the Ethics Committee for Animal Experiments of Jinan University.

### MTT Reduction Assay

The tetrazolium salt 3-(4,5-dimethylthiazol-2-yl)-2,5-diphenyltetrazolium bromide dye (MTT) assay was used to assess neurotoxicity. The assay was performed according to the procedure described in our previous work (Chen H.Y. et al., 2015). Briefly, neurons were cultured in 96-well plates for 8 DIV. Treatments of compound **22a** (0.1, 1, 10, 100 μM), TMP (100 μM) and memantine (5 μM) were used as controls for 2 h before 200 μM glutamate was added to the media. To determine the possible pathways involved in the effect of compound **22a**, CGNs were pretreated with 1 μM LY294002 (a PI3K inhibitor) or 1 μM Akt-iv (an Akt inhibitor) for 30 min before administration of compound **22a** (10 μM), and were then challenged by glutamate. After 24 h, the media of each well was supplemented with 10 μL of 5 mg/mL MTT, and the plate was put in an incubator at 37°C for 4 h under humidified conditions. A microplate reader was used to measure the absorbance of the samples at 570 nm wavelength.

### Lactate Dehydrogenase (LDH) Release

The activity of LDH released into the incubation medium was used as an indicator to determine cellular injury. CGNs were pretreated with compound **22a** (0.1, 1, 10, 100 μM) or TMP (100 μM) and memantine (5 μM) for 2 h, then 200 μM glutamate was added to incubated for 24 h. The cytotoxicity detection kit (Roche Applied Science, Germany) was used to determine LDH activity. LDH release was calculated according to the manufacturer’s instructions. A microplate reader was used to measure the absorbance at 490 nm wavelength. Cytotoxicity (%) was calculated with the following equation:

$$\begin{array}{r} \text{Cytotoxicity}(\%)=(\text{experimental LDH release}-\\ \text{spontaneous LDH release})/(\text{Maximum LDH release}-\\ \text{spontaneous LDH release}{)}^{*}\text{100} \end{array}$$

Experimental LDH release represents the activity of released LDH in cells pretreated with test compounds and glutamate, Spontaneous LDH release represents the activity of released-LDH in untreated normal cells, and Maximum LDH release represents maximum LDH activity determined by lysing the cells (100% dead cells) with Triton X-100 (final concentration 2% for 4 h at 37°C). All assays were performed in six-replicates and repeated three times.

### Hoechst Staining

Chromatin condensation was detected by nucleus staining with Hoechst 33342 (Beyotime, China) as previously described (Chen H.Y. et al., 2015). CGNs of 4–5 × 10^5^ cells/well were cultured in a 24-well plate for 8 DIV. Pretreatment of compound **22a** (100 μM) and memantine (5 μM) as the positive control at the indicated concentrations for 2 h before 200 μM glutamate was added into the media. After 24 h, cells were washed with ice-cold phosphate-buffered saline (PBS) and fixed with 4% formaldehyde in PBS. Hoechst 33342 (5 mg/mL) was added to the media for 5 min at 4°C. Samples were observed and photos were taken under a fluorescence microscope. The fluorescence images were photo-taken from three different fields of each experiment with a total of three independent experiments. The number of apoptotic nuclei was counted and expressed as a percentage of total 100 nuclei counted/field.

### Measurement of Intracellular Reactive Oxygen Species (ROS)

Cerebellar granule neurons were cultured in 96-well plates at a density of 1.0–1.5 × 10^5^ cells/well for 8 DIV. After pretreatment with compound **22a** (0.1, 1, 10, 100 μM) or memantine (5 μM) for 2 h, they were then exposed to 200 μM glutamate for 24 h. The total intracellular ROS were detected using H_2_DCF-DA (10 μM), and a microplate reader was used to measure the fluorescence intensity. The fluorescence intensity/per well was normalized against the MTT absorption value of the same well. The fluorescence values of the treated group were calculated as a percentage of the fluorescence of the control cells.

### Measurement of Mitochondrial Membrane Potential

Cerebellar granule neurons were placed in a 96-well plate at a density of 1.0–1.5 × 10^5^ cells/well. After 8 DIV, compound **22a** or memantine was added to pretreat cells at the indicated concentrations for 2 h. After exposure to 200 μM glutamate for 24 h, the cells were washed three times and then stained with 2 μM JC-1 (Beyotime, China), a molecular probe to measure mitochondrial membrane potential (MMP), for 10 min. The microplate reader was used to measure the fluorescence intensity using 490 nm/530 nm excitation and 525 nm/590 nm dual emissions. The mitochondrial accumulation of JC-1 is dependent upon MMP, which is calculated as a decrease in the ratio of 590 nm to 525 nm emissions.

### Measurement of Intracellular ATP Levels

Cerebellar granule neurons were placed in a 96-well plate at a density of 1.0–1.5 × 10^5^ cells/well. After 8 DIV, **22a** or memantine was added to pretreat at the indicated concentrations for 2 h. After the following incubation with 200 μM glutamate for 24 h, a ATP Assay Kit was used to detect the intracellular ATP levels by using the (Beyotime, China) according to the manufacturer’s protocol. The intracellular ATP levels of the treated group were normalized to the control cells’.

### Western Blot Assay

Cerebellar granule neurons were cultured in a 6-well plate at a density of 2 × 10^6^ per well in 1.5 mL. At 8 DIV, cells were pretreated with **22a** for 2 h before incubation with 200 μM glutamate for another 12 h. Cells were harvested in a cell lysis buffer supplemented with 1% PMSF (phenylmethanesulfonyl fluoride) as well as 1% protease inhibitor cocktail (Roche Applied Science). The cellular protein concentrations were determined by the BCA assay (Pierce, Rockford, IL, United States) after incubation for 15 min on ice and centrifugation (14,000 *g*) for 10 min at 4°C. SDS sample buffer was added to dilute the cell lysates, and the mixture was heated for 5 min at 100°C. The protein (30 μg) was separated on a 10% SDS–polyacrylamide gel. After transferring protein to polyvinyldifluoride membranes, 5% BSA was used as a blocking buffer to block the membranes. After incubation with the primary antibodies at 4°C overnight, signals were attained by binding a secondary antibody conjugated to horseradish peroxidase. Blots were detected using the chemiluminescence enhancer detection ECL plus kit (Fude Biological Technology Co., Ltd., China) and images captured using a detecting system (Carestream Health, United States). Quantitation of the digitalized images was analyzed based on their mean pixel density by using Carestream software system, and described as an expression ratio of target protein to β-actin (a loading control protein).

### Silencing of PGC1α by Small Interfering RNA (siRNA)

Cerebellar granule neurons at 8 DIV were transfected with siRNA using transfection reagent Opti-MEM I (Invitrogen, Carlsbad, CA, United States) and Lipofectamine2000 (Invitrogen) according to the manufacturer’s instructions and the previous studies’ (Kim et al., 2014). Experiments were carried out 24 h after the cells were transfected with PGC1α siRNA or scrambled siRNA.

### PGC1α Transcriptional Activity

PC12 cells were plated in 24-well plates and transfected with a PGC1α luciferase reporter plasmid along with Renilla luciferase control vector by using the Amaxa Nucleofector II, which can be used for transfection of any DNA vectors (expression plasmids, shRNA vectors) into difficult-to-transfect cell lines and primary cells. Then 12 h after transfection, the cells were treated with compound **22a** at the indicated concentrations for 24 h. Cells were harvested and analyzed using a Dual-Glo luciferase assay kit (Promega, Madison, WI, United States) following the manufacturer’s instructions. Firefly luciferase activity was normalized to Renilla luciferase activity.

### Immunofluorescence

Cerebellar granule neurons of 4–5 × 10^5^ cells/well were cultured in a 24-well plate for 8 DIV, and were treated with compound **22a** (100 μM). After 12 h, cells were washed with ice-cold phosphate-buffered saline (PBS) and fixed with 4% formaldehyde in PBS for 30 min at room temperature. The cells were washed with ice-cold PBS containing 5% BSA acting as a blocking buffer for 1 h at room temperature before being incubated with anti-Nrf2 antibody (1:500) overnight at 4°C. The cells were then washed with ice-cold PBS and incubated with FITC antibody (1:200) (Sigma-Aldrich, St. Louis, MO, United States) containing 200 nM DAPI for 2 h at room temperature. Samples were observed and photos were taken under a fluorescence microscope.

### Statistical Analysis

All experiments were carried out at least three times with different neuronal preparations, data from which was presented as means ± SEM. Analysis of variance (ANOVA) and Bonferroni’s post-test were used for statistical comparisons, with *P* < 0.05 being considered as statistical significance.

## Results

### Compound 22a Effectively Prevents Glutamate-Induced Neurotoxicity

**FIGURE 1:**
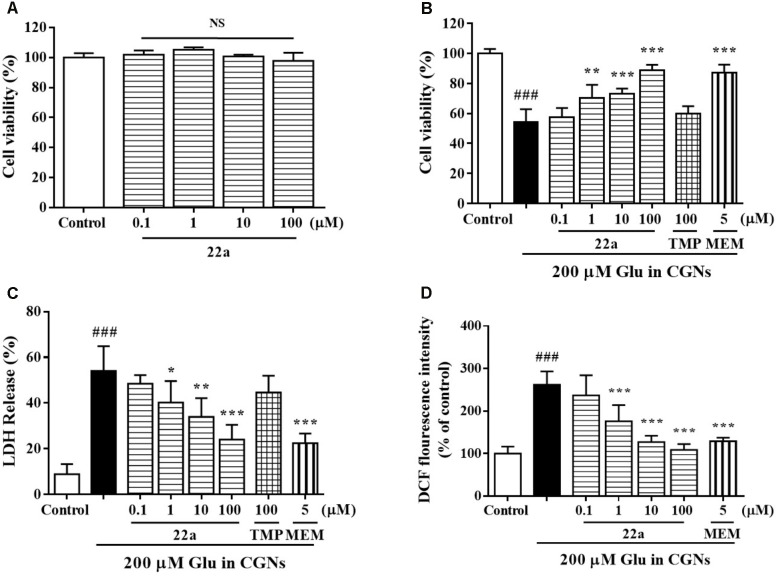
Compound **22a** prevents glutamate-induced neurotoxicity. **(A)** Toxicity of compound **22a** on CGNs. CGNs were pre-incubated with compound **22a** for 24 h. Cell viability was measured using the MTT assay. **(B)** Compound **22a** prevents glutamate-induced neuronal death in CGNs. CGNs were pre-incubated with various agents for 2 h, then exposed to 200 μM glutamate. Cell viability was measured at 24 h post glutamate challenge using the MTT assay. **(C)** Compound **22a** prevents glutamate-induced increase of LDH in CGNs (% of control). CGNs were pre-incubated with various agents for 2 h, then exposed to 200 μM glutamate. LDH release was measured at 24 h after glutamate challenge. **(D)** Compound **22a** attenuates glutamate-induced increase of ROS in CGNs. CGNs were pre-incubated with various compounds for 2 h, then exposed to 200 μM glutamate for another 24 h. Intracellular hydrogen peroxide was measured by DCF-DA. Data were expressed as the mean ± SEM of three separate experiments; ^###^*p* < 0.001 versus control group; ^∗^*p* < 0.05, ^∗∗^*p* < 0.01, and ^∗∗∗^*p* < 0.001 versus glutamate group. Ctrl means control. Glu, glutamate. MEM, memantine.

At 8 DIV, CGNs were exposed to increasing concentrations of glutamate, which induced a concentration-dependent neuronal death from 50 to 1000 μM (data not shown). Since low doses of glutamate induced apoptosis instead of necrosis in primary CGNs (Du et al., 1997), a 200 μM glutamate concentration that produced approximately 54% neuronal loss was used for the subsequent experiments. Treatment with compound **22a** up to 100 μM for 24 h didn’t cause any cytotoxicity to CGNs (**Figure 1A**). Pretreatment with serial concentrations of compound **22a** (1–100 μM) could prevent glutamate-induced toxicity and the maximum cell viability reached 88.7% at 100 μM, similar to that of positive control memantine (87.1%) and higher than that of TMP (60.0%) (**Figure 1B**). The neuroprotection of compound **22a** against glutamate triggered cytotoxicity was more potent than that of TMP.

To further confirm the protection of compound **22a** against glutamate-induced neurotoxicity, we measured the LDH release and intracellular ROS production in CGNs. Compound **22a** and memantine significantly prevented glutamate-induced LDH release. TMP (100 μM) had a marginal effect in this model (**Figure 1C**). Furthermore, pretreatment with compound **22a** from 1 to 100 μM and memantine significantly decreased the intracellular ROS overproduction caused by glutamate in CGNs (**Figure 1D**).

### Compound **22a** Inhibits Glutamate-Induced Cellular Apoptosis in CGNs

**FIGURE 2:**
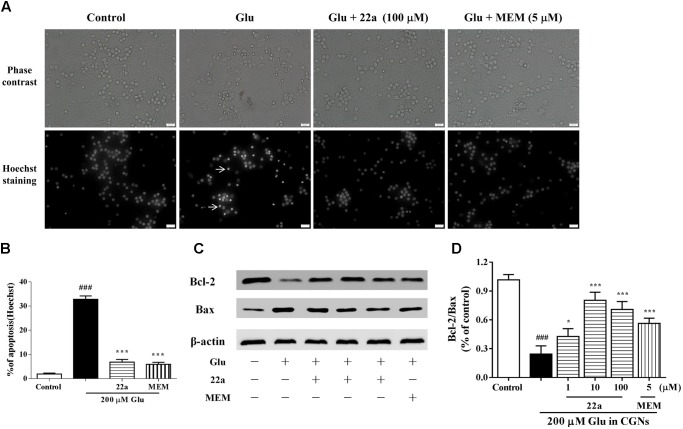
**22a** inhibits glutamate-induced cell apoptosis in CGNs. **(A)** Compound **22a** prevents glutamate-induced increase of pyknotic nuclei in CGNs. CGNs were pre-incubated with various agents for 2 h, then exposed to 200 μM glutamate. CGNs were stained with Hoechst 24 h after the glutamate challenge. Photographs were from a representative experiment; experiments were repeated three times (original magnification 400×). **(B)** Statistical analysis of the number of pyknotic nuclei. The number of pyknotic nuclei with condensed chromatin was counted from representative Hoechst staining photomicrographs and is represented as a percentage of the total number of nuclei counted. **(C)** Western blot of apoptosis related proteins Bcl-2 and Bax. **(D)** Densitometry analysis of protein expression ratios of Bcl-2 and Bax. Data were expressed as the mean ± SEM of three separate experiments; ^###^*p* < 0.001 versus control group; ^∗^*p* < 0.05 and ^∗∗∗^*p* < 0.001 versus glutamate group.

It has been reported that a 200 μM glutamate concentration induced neuronal damage via apoptosis (Du et al., 1997). Compound **22a** and memantine significantly reversed the cell counts of nuclear condensation induced by glutamate (**Figures 2A,B**). In **Figure 2C**, two apoptosis related proteins, Bcl-2 and Bax, were determined by Western blot analysis. It was found that pretreatment with compound **22a** reversed the Bcl-2 down-regulation and Bax up-regulation induced by glutamate. The Bcl-2/Bax ratio was increased by pretreatment with compound **22a** in a concentration-dependent manner (**Figure 2D**).

### Compound **22a** Improves the Mitochondrial Dysfunction Induced by Glutamate

**FIGURE 3:**
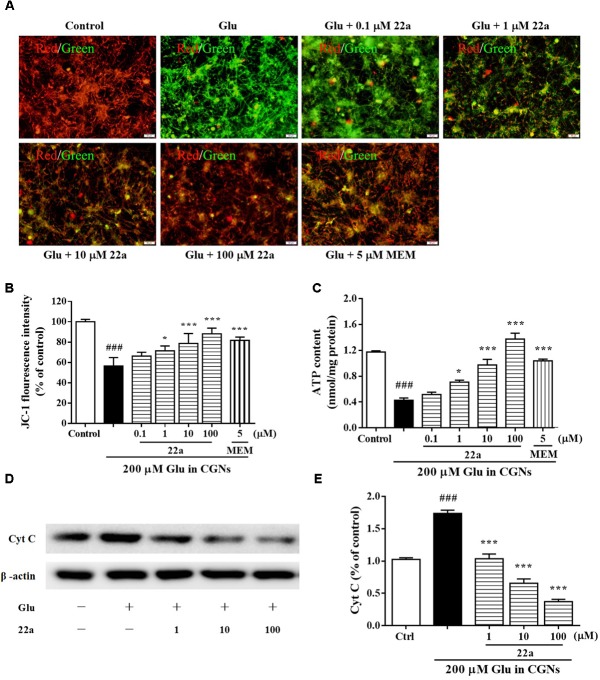
Compound 22a improves mitochondrial dysfunction induced by glutamate. **(A)** Compound **22a** prevents glutamate-induced changes of MMP in CGNs. CGNs were pre-incubated with various agents for 2 h, then exposed to 200 μM glutamate. The MMP was evaluated by staining with the potential sensor JC-1. The fluorescence shift from red to green was detected by a microplate reader. **(B)** Quantitative analysis of the red fluorescence to green fluorescence (590 nm/529 nm) ratio in **(A)**. **(C)** Compound **22a** attenuates glutamate-induced ATP depletion in CGNs. **(D)** Immunoblot assay was performed with antibodies against Cyt C. **(E)** Densitometric analysis of **(D)**. Data were expressed as the mean ± SEM of three separate experiments; ^###^*p* < 0.001 versus control group; ^∗^*p* < 0.05, ^∗∗^*p* < 0.01, and ^∗∗∗^*p* < 0.001 versus glutamate group.

To examine whether the neuroprotective effects of compound **22a** was due to preservation of mitochondrial function, we measured the MMP collapse and mitochondrial ATP release. Changes of MMP were monitored by a JC-1 molecular probe, and the fluorescence shift from red to green reflected the depolarization of MMP (Chaoui et al., 2006). It was found that pretreatment with compound **22a** concentration-dependently and strongly restored MMP decrease (red fluorescence/green fluorescence) in glutamate-treated CGNs (**Figures 3A,B**). We also found that glutamate induced a significant decrease in mitochondrial ATP production, whereas pretreatment with compound **22a** significantly prevented glutamate-induced decreases in ATP levels (**Figure 3C**). Memantine was also effective ameliorating the decrease of MMP and ATP in glutamate-treated CGNs. In addition, when Cyt C release was measured by Western blot as shown in **Figures 3D,E**, it was found that **22a** concentration-dependently prevented glutamate-induced Cyt C release.

### Compound **22a** Up-Regulates PGC1α/Nrf2 Pathway Through Activation of PI3K/Akt

**FIGURE 4:**
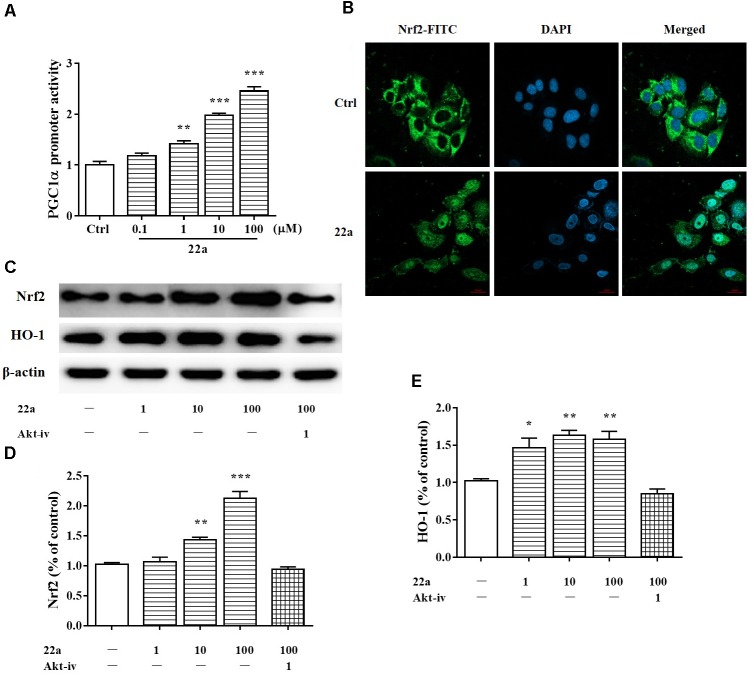
Compound **22a** activates of PGC1α/Nrf2 signaling pathway. **(A)** Compound **22a** (0.1, 1, 10, 100 μM) increased the transcriptional activity of PGC1α determined by the luciferase reporter assay. **(B)** Compound **22a** (100 μM) induced the nuclear translocation of Nrf2 determined by immunofluorescence staining. Left panel: green fluorescence showing Nrf2 localization. Middle panel: stained nucleus with DAPI. Right panel: merged images from green and blue filters. Scale bar: 20 μm. **(C)** Immunoblot assay was performed with antibodies against Nrf2 and HO-1. **(D,E)** Densitometric analysis of the protein expression in **(C)**. CGNs were treated with compound **22a** (1, 10, 100 μM) for 12 h. Data were expressed as the mean ± SEM of three separate experiments; ^∗^*p* < 0.05, ^∗∗^*p* < 0.01, and ^∗∗∗^*p* < 0.001 versus control group.

PGC1α was considered to be a major regulator of mitochondrial biogenesis. It is thought to regulate the expression of Nrf2 (Wareski et al., 2009; Robinson et al., 2014), a major transcription factor against oxidative stress (Nguyen et al., 2009). As shown in **Figure 4A**, Compound **22a** increased the transcriptional activity of PGC1α in a concentration-dependent manner. Nrf2 nuclear translocation was further investigated with PC12 cells after treatment with 100 μM compound **22a** for 12 h. As shown in **Figure 4B**, **22a** pretreatment increased Nrf2 accumulation in the nucleus. Nrf2 is a crucial regulator against oxidative stress and it is of interest to investigate the involvement of HO-1 protein expression. As shown in **Figures 4C–E**, CGNs treated with compound **22a** concentration-dependently increased Nrf2 and HO-1 expression. PI3K/Akt activation was reported to contribute to the up-regulation of the Nrf2 signal (Lee et al., 2015). We assessed if compound **22a**-induced Nrf2 and HO-1 expression was affected by Akt inhibitor Akt-iv. As shown in **Figures 4C–E**, compound **22a**-induced up-regulation of Nrf2 and HO-1 was completely abolished by Akt-iv.

### Compound **22a** Activates PGC1α/Nrf2 Signaling Pathway in CGNs Treated With Glutamate

**FIGURE 5:**
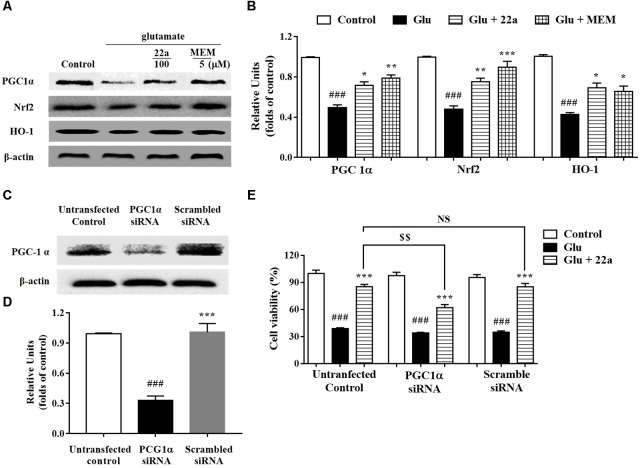
Involvement of the PGC1α/Nrf2 pathway in neuroprotection exerted by compound **22a** in CGNs. **(A)** Representative blots showed the expression of proteins PGC1α, Nrf2 and HO-1 in CGNs after different treatments. **(B)** Densitometric analysis of the protein expression in **(A)**. **(C)** Representative blots showed the protein expression of PGC1α was silenced in CGNs pretreated with PGC1α siRNA, but not when pretreated with scrambled siRNA. **(D)** Densitometric analysis of **(C)**. **(E)** PGC1α siRNA significantly attenuated the neuroprotective effects of compound **22a** against glutamate-induced neuronal cell death in CGNs. CGNs were transfected with either PGC1α siRNA or scrambled siRNA for 24 h and then pretreated with compound **22a** (10 mM) for 2 h before exposure to 200 μM glutamate. Cell viability was measured at 24 h post glutamate exposure using the MTT assay. Data were expressed as the mean ± SEM of three experiments; ^###^*p* < 0.01 versus control group; ^∗^*p* < 0.05, ^∗∗^*p* < 0.01, and^∗∗∗^*p* < 0.001 versus glutamate group; NS means no significance; ^$$^*p* < 0.01 versus **22a** in the not-transfected control group.

To further elucidate whether the PGC1α/Nrf2 signaling pathways were involved in the protective effect of compound **22a** against glutamate-induced neuron injury, the two protein’s expression levels were examined with Western blotting. We found that glutamate significantly decreased PGC1α, Nrf2, and HO-1 protein expression (**Figures 5A,B**); however, compound **22a** pretreatment reversed these decreases. To confirm the role of PGC1α activation in the neuroprotective effects of compound **22a**, we tested the blocking effect of PGC1α siRNA transfection. We found that PGC1α siRNA transfection dramatically decreased PGC1α protein expression (**Figures 5C,D**). In cell viability tests, PGC1α siRNA transfection considerably attenuated the neuroprotection of compound **22a**; however, a scrambled RNA transfection that was used as a negative control did not yield any significant effects on PGC1α expression or on cell viability (**Figure 5E**).

### Compound **22a** Reversed the Inhibition of PI3K/Akt/GSK3β Pathway Caused by Glutamate

**FIGURE 6:**
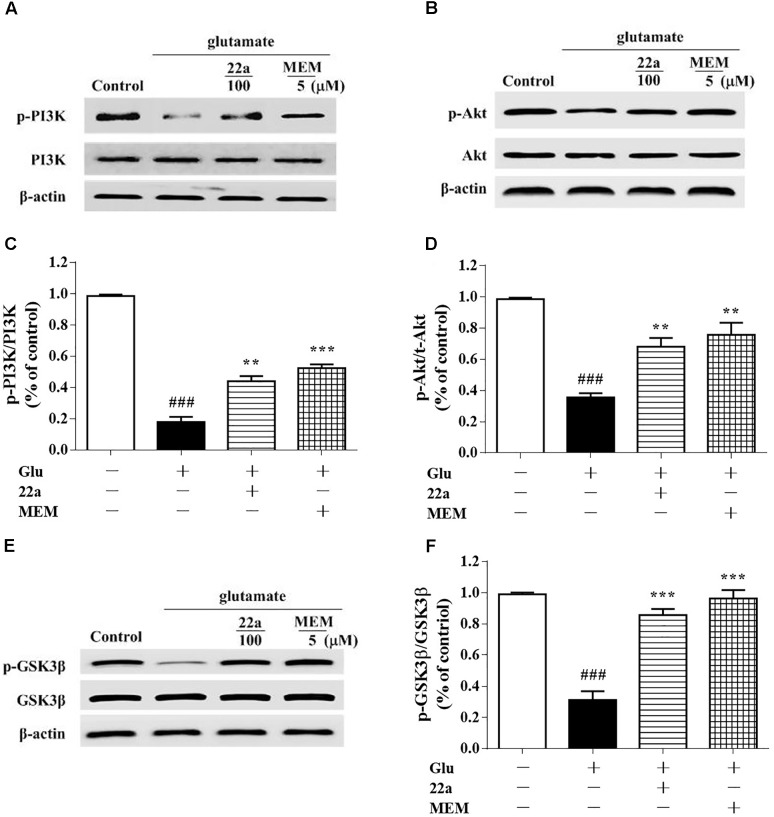
Compound **22a** activates PI3K/Akt pathway in glutamate treated CGNs. **(A,C,E)** Representative blots showed the protein expression of p-PI3K/PI3K **(A)**, p-Akt/Akt **(C)**, and p-GSK3β/GSK3β **(E)** in CGNs. CGNs were pretreated with compound **22a** and memantine for 2 h before exposure to glutamate. **(B,D,F)** Densitometric analysis of the protein expression in **(A,C,E)**. Data were expressed as the mean ± SEM of three experiments; ^###^*p* < 0.001 versus control group; ^∗∗^*p* < 0.01 and^∗∗∗^*p* < 0.001 versus glutamate treatment group.

To investigate the signaling pathways involved in the protective effects of compound **22a** against glutamate-caused excitotoxicity, we evaluated the correlation between cell viability and PI3K/Akt activation. The levels of p-PI3K, p-Ser473-Akt and p-Ser9-GSK3β were analyzed by Western blot. As shown in **Figures 6A–D**, glutamate down-regulated the phosphorylation of PI3K and Akt, while pretreatment with both compound **22a** and memantine reversed the suppressed phosphorylation of PI3K and Akt. GSK-3β is the molecule downstream of PI3K/Akt and is phosphorylated by the PI3K/Akt pathway. As shown in **Figures 6E,F**, there was a significant increase in the phosphorylation of GSK-3β after treatment with compound **22a** in the presence of glutamate.

### PI3K and Akt Inhibitors Attenuate the Neuroprotective Effects of Compound **22a**

**FIGURE 7:**
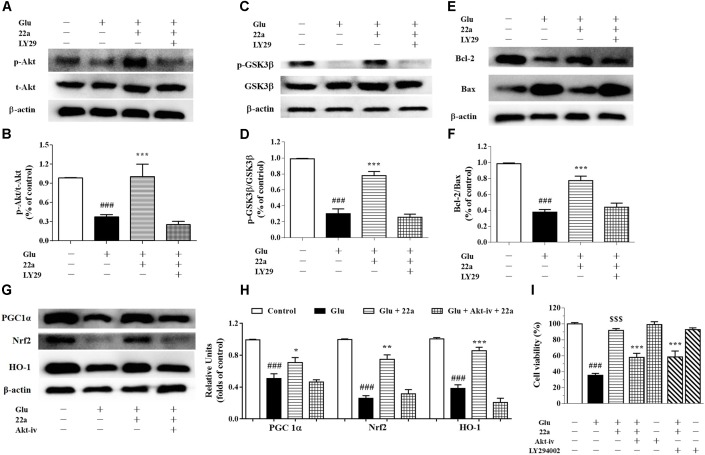
Involvement of the PI3K/Akt pathway in neuroprotection exerted by compound **22a** in CGNs. **(A,C,E)** Representative blots showed the protein expression of p-Akt/Akt **(A)**, p-GSK3β/GSK3β **(C)**, Bcl-2 and Bax **(E)**. CGNs were pretreated with 1 μM LY294002 for 30 min before administration of compound **22a** (10 mM), followed by glutamate challenge. **(B,D,F)** Densitometric analysis of the protein expression in **(A,C,E)**. **(G)** Representative blots showed the protein expression of PGC-1α, Nrf2 and HO-1. **(H)** Densitometric analysis of the protein expression in **(G)**. **(I)** Pretreatment with an Akt inhibitor (Akt-iv) and a PI3K inhibitor (LY294002) attenuated the protective effects of compound **22a** on glutamate-treated CGNs. Data were expressed as the mean ± SEM of three experiments; ^###^*p* < 0.001 versus control group; ^∗^*p* < 0.05, ^∗∗^*p* < 0.01, and^∗∗∗^*p* < 0.001 versus glutamate treatment group; ^$$$^*p* < 0.001 versus “**22a**+glutamate” treatment group. LY29 refers to PI3K inhibitor LY294002.

Activation of the pro-survival PI3K/Akt signaling pathway has been shown to be important for neuroprotection (Cantrell, 2001). Consistent with our previous study (Xu et al., 2016), when exposing the cells to a PI3K inhibitor, LY294002 (1 μM), 30 min prior to the addition of compound **22a**, phosphorylation of Akt and GSK3β was nearly completely blocked by LY294002 as shown in **Figures 7A–D**. Pretreatment with LY294002 reversed compound **22a**-prompted Bcl-2 up-regulation and Bax down-regulation; the Bcl-2/Bax ratio was decreased by LY294002 (**Figures 7E,F**). In line with previous reports that PI3K/Akt is involved in Nrf2 mediated HO-1 expression (Lee et al., 2015), when cells were exposed to an Akt inhibitor, Akt-iv (1 μM), 30 min prior to **22a** treatment, the up-regulation of PGC1α, Nrf2 and HO-1 proteins’ expression by compound **22a** was almost completely suppressed by Akt-iv, as shown in **Figures 7G,H**.

To further confirm the involvement of the PI3K/Akt pathway in the neuroprotection exerted by compound **22a** in CGNs damaged by glutamate, a specific PI3K inhibitor LY294002 and an Akt inhibitor Akt-iv were applied in a cell viability assay. LY294002 and Akt-iv significantly attenuated the neuroprotection of compound **22a** against glutamate toxicity (**Figure 7I**).

## Discussion

Glutamate is the principal excitatory amino acid neurotransmitter with complex biological activities (Pita-Almenar et al., 2006; Paoletti, 2011). However, a high concentration of extracellular glutamate is toxic to nerve cells and is considered to be a key contributor in the pathogenesis of neurodegenerative diseases such as ischemic stroke (Wahl et al., 1994). In our previous study, we reported that compound **22a** exhibited neuroprotective effects against oxidative stress-induced neuronal loss *in vitro* and protected against ischemic stroke *in vivo* (Chen et al., 2017). However, the exact mechanisms underlying the neuroprotection of compound **22a** is still unknown. Therefore, the neuroprotective effects of compound **22a** against glutamate-induced excitotoxicity were investigated in the current study. We demonstrated that compound **22a** protected against glutamate-induced neurotoxicity in CGNs. Meanwhile, we found that compound **22a** reversed the MMP collapse and alternation of Bcl-2 and Bax expression to attenuate glutamate-induced cellular apoptosis. Our research further demonstrated that the neuroprotective effects of compound **22a** were intermediated by the stimulation of PI3K/Akt and PGC1α/Nrf2 pathways.

Glutamate is one of the pathological factors in cerebral ischemic disease, and can cause cell apoptosis and MPP reduction, both of which are initiated by the interaction between pro- and anti-apoptotic Bcl-2 family members (Chen Q. et al., 2015). In addition, glutamate toxicity induces mitochondrial dysfunction. Mitochondria are recognized as a center of intracellular energy metabolism, and mitochondrial Ca^2+^ is a positive effector of ATP synthesis (Feissner et al., 2009). Ca^2+^ overload, however, results in free radical generation and mPTP opening, which in turn causes mitochondrial depolarization, matrix solute loss, and Cyt C release (Bernardi and Rasola, 2007). Moreover, the overproduction of ROS is also reported to be related to mPTP opening (Christophe and Nicolas, 2006). In our study, compound **22a** pretreatment significantly prevented intracellular ATP reduction and ROS aggregation, and mitigated MMP dissipation and Cyt C release. Our data further uncovered that compound **22a** reversed the up-regulation of Bax and down-regulation of Bcl-2 expression induced by glutamate. Furthermore, the expression of Bcl-2, an integral membrane protein, is recognized as a hallmark of cell death associated with mitochondria dysfunction (Wallgren et al., 2013). Altogether, these results imply that increased Bcl-2 expression may represent an endogenous repair mechanism against apoptotic pathway, indicating that **22a** may block the Bax-mediated decrease of MMP or promote mitochondrial homeostasis against glutamate-caused CGNs damage.

As described above, mitochondria play a vital role in many fundamental cellular processes, ranging from energy production and metabolism to apoptosis (Robinson et al., 2014). PGC1α is a transcriptional co-activator that regulates the transcription of numerous genes involved in cellular metabolism, including mitochondrial biogenesis and respiration and ROS metabolism (Shin et al., 2011). PGC1α is also a potent stimulator of mitochondrial respiration and gene transcription that acts by activating nuclear respiratory factors Nrf1 and Nrf2, which in turn regulate expression of mitochondrial transcription factor A (Tfam) and other nuclear-encoded mitochondrial proteins (Wareski et al., 2009). Numerous studies indicate that Nrf2 combines with the promoter of ARE to create general antioxidant responses, which is recognized as a promising method to therapeutically reestablish the CNS redox balance in neurodegenerative disorders (Lim et al., 2014). Among the enzymes that are redox-sensitive inducible is HO-1, which can protect neurons from acute insults under stress conditions thanks to its antioxidant and anti-inflammatory properties (Chen, 2014). In line with previous studies, we found that compound **22a** could enhance PGC1α transcriptional activity and induce nuclear accumulation of Nrf2 in PC12 cells (**Figures 4A,B**). In addition, we found that glutamate treatment significantly down-regulated the protein expression of PGC1α and pretreatment with compound **22a** significantly reversed the down-regulated expressions of PGC1α, Nrf2 and HO-1 induced by glutamate in CGNs (**Figures 5A,B**). Several investigations present that Nrf2 is tightly regulated in neurons through signaling pathways such as PI3K/Akt, which is reported to have a Nrf2-dependent role in activating HO-1 expression (Wang et al., 2008; Zhang et al., 2012; Yin et al., 2015). In this sense, the present study was designed to investigate whether the PI3K/Akt pathway is involved in regulating the PGC1α and Nrf2/HO-1 activation resultant from compound **22a**’s presence, and what effect that has on compound **22a**’s subsequent protective against glutamate-induced neurotoxicity. Our results demonstrated that a specific Akt inhibitor significantly suppressed the enhanced expression of PGC1α, Nrf2 and HO-1 induced by compound **22a** (**Figures 7G,H**). Moreover, to some extent, knockdown of PGC1α was found to reverse the neuro-protective effect of compound **22a** against toxic stress (**Figures 5C–E**). As such, compound **22a** may pave an effective and practical way to modulate PGC1α activity in neurons.

The PI3K/Akt pathway plays a critical role in preventing the neuronal cell death seen in hypoxic and excitotoxic conditions *in vitro*. Inhibiting the PI3K/Akt pathway exacerbates ischemic neuronal death (Lai et al., 2014). Previous reports indicate that neuroprotective strategies against glutamate-induced excitotoxicity in the cortex and hippocampus of postnatal brains can trigger the activation of PI3K/Akt (Shah et al., 2014). Additionally, Akt activation is capable of suppressing several pro-apoptotic proteins, including members of the Bcl-2 family and some signaling molecules such as GSK-3β (Maurer et al., 2014). In our study, phosphorylation of PI3K, Akt and GSK3β in CGNs was significantly down-regulated when cells were exposed to glutamate; however, compound **22a** pretreatment reversed these changes (**Figures 6A–F**). In addition, PI3K inhibitor LY29004 significantly inhibited the up-regulation of phosphorylated Akt and GSK3β expression while reversing compound **22a**-induced up-regulation of Bcl-2 and down-regulation of Bax expression (**Figures 7A–F**). Importantly, PI3K and Akt inhibitors completely abolished the neuroprotection conferred by compound **22a** pretreatment (**Figure 7I**). Summarily, our results indicate that PI3K/Akt pathway activation is involved in the neuro-protection of compound **22a** against CGNs injury induced by glutamate.

In summary, compound **22a** effectively prevented glutamate-induced excitotoxicity of CGNs via involvement of the PI3K/Akt and PGC1α/Nrf2 pathways. Our results suggest that compound **22a** might be of benefit in preventing neuronal death from ischemic stroke.

## Ethics Statement

All animal studies were conducted following to the handbook of the Experimental Animal Care and Use Committee of Jinan University, and the experimental protocols were approved by the Ethics Committee for Animal Experiments of Jinan University.

## Author Contributions

HC conceptualized and designed the experiments, performed the research, analyzed and interpreted the results, and wrote the manuscript. JC and ZyZ helped design and performed the experiments. YW, ZjZ, YS, GZ, LS, and PY conceptualized and designed the experiments, analyzed and interpreted the results, and revised the manuscript.

## Conflict of Interest Statement

The authors declare that the research was conducted in the absence of any commercial or financial relationships that could be construed as a potential conflict of interest.

## Abbreviations

Akt: protein kinase B
CGN: cerebellar granule neurons
Cyt C: cytochrome c
GSK3β: glycogen synthase kinase 3β
HO-1: hemooxygenase 1
MMP: mitochondria membrane potential
Nrf2: transcriptional factor NF-E2-related factor 2
PGC1α: peroxisome proliferator-activated receptor gamma coactivator 1-alpha
PI3K: phosphatidylinositol 3-kinase
ROS: reactive oxygen species
TMP: tetramethylpyrazine
